# Glyphosate vs. Glyphosate-Based Herbicides Exposure: A Review on Their Toxicity

**DOI:** 10.3390/jox12010003

**Published:** 2022-01-17

**Authors:** Carlos Martins-Gomes, Tânia L. Silva, Tatiana Andreani, Amélia M. Silva

**Affiliations:** 1 Centre for Research and Technology of Agro-Environmental and Biological Sciences (CITAB), University of Trás-os-Montes and Alto Douro (UTAD), 5001-801 Vila Real, Portugal; tanialfs10@gmail.com (T.L.S.); tatiana.andreani@fc.up.pt (T.A.); 2 Department of Biology and Environment, School of Life Sciences and Environment, University of Trás-os-Montes and Alto Douro (UTAD), 5001-801 Vila Real, Portugal

**Keywords:** glyphosate, AMPA, glyphosate-based herbicides, xenobiotics, animal models, metabolism, toxicity

## Abstract

Glyphosate-based herbicide has been the first choice for weed management worldwide since the 1970s, mainly due to its efficacy and reported low toxicity, which contributed to its high acceptance. Many of the recent studies focus solely on the persistence of pesticides in soils, air, water or food products, or even on the degree of exposure of animals, since their potential hazards to human health have raised concerns. Given the unaware exposure of the general population to pesticides, and the absence of a significant number of studies on occupational hazards, new glyphosate-induced toxicity data obtained for both residual and acute doses should be analyzed and systematized. Additionally, recent studies also highlight the persistence and toxicity of both glyphosate metabolites and surfactants present in herbicide formulations. To renew or ban the use of glyphosate, recently published studies must be taken into account, aiming to define new levels of safety for exposure to herbicide, its metabolites, and the toxic excipients of its formulations. This review aims to provide an overview of recent publications (2010–present) on in vitro and in vivo studies aimed at verifying the animal toxicity induced by glyphosate, its metabolite aminomethylphosphonic acid (AMPA) and glyphosate-based formulations, evaluated in various experimental models. Apart from glyphosate-induced toxicity, recent data concerning the role of surfactants in the toxicity of glyphosate-based formulations are discussed.

## 1. Introduction

Over the years, the dependence of agricultural activities on herbicides has been an environmental and public health issue with a difficult resolution. Driven by population growth and the need to produce an increasing amount of food products, herbicide use proved to be the quickest and cheapest solution, by avoiding hand weeding, a process that was slower and required more human resources, in addition to reducing fuel costs for machinery [1]. Although the use of herbicides on a larger scale started mainly with inorganic compounds in the final quarter of the 19th century (after 1874), for example, using iron and copper sulfates, during the 20th century the impact of herbicide use gained a new dimension with the introduction of new synthetized compounds such as atrazine, bromacil, paraquat and glyphosate, between 1952 and 1971 [2].

These compounds are usually classified by their herbicidal mode of action and their molecular targets [3]. Atrazine, a member of triazines family, is a photosynthesis inhibitor. It interacts with the D1 protein of plant cells’ photosystem II (PSII), impairing the electron transport chain [4]. The PSII is also bromacil’s molecular target [5], while the main target of paraquat is photosystem I (PSI), as the herbicide acts as a single electron acceptor, redirecting electrons heading to PSI. This reaction initiates a chain of oxidative events leading to the formation of superoxide radical [6]. In fact, half of the herbicides available in the market are inhibitors of photosystems and electron transport chain [5]. Nevertheless, among the >200 compounds available on the market with herbicidal properties, the molecular target of such activity is unknown for most of them, and within the 29 modes of action described, the inhibition of acetolactate synthase, hydroxyphenylpyruvate dioxygenase, acetyl-coenzyme A carboxylase, protoporphyrinogen oxidase and phytoene desaturase, together with the PSI and PSII inhibition, are the most common targets for most products [7].

The increase in herbicide use over the last century is well documented in many developed countries, such as North America, South America, Europe and Asia. Reports from 2014 stated that herbicides market generated USD 17 billion annually, with growth projections. In China, for example, the pesticide-treated land area rose from 1 to 70 million ha in 35 years (from 1970 to 2005) [1,7]. According to more recent evaluations, herbicides represent almost USD 24 billion in market value, a value expected to increase USD 10 billion by 2022 [8]. Contributing greatly to this market value, North and South American countries, mainly the United States of America, Brazil, Argentina and Canada, present the highest share of global herbicide use. In Europe, Russia (and previously USSR), France and Ukraine are listed as the major consumers [8]. Nevertheless, the common and unregulated use of easily obtainable herbicides is a major issue, as their toxicity is frequently observed in animals, and their persistency in soil, water, and atmosphere has also been reported. Mentioned above, atrazine is noted as one of the most used herbicides worldwide; its presence has been confirmed in air, fog, ice, seawater, freshwater and rain [9,10]. Concerning its effect on human health, atrazine acts as a potential endocrine disruptor [10,11], modulates the cell cycle and the cell growth, affects the intestinal epithelium transport regulation, and presents liver cytotoxicity and genotoxicity [10,12,13,14,15]. These reasons were behind the ban applied by the European Union to this chemical in 2004 [9].

Another example is paraquat-induced toxicity, which has been widely described (e.g., [16,17]). Although presenting slow skin and oral absorption, its bioaccumulation in human organs, namely in lung or in kidney, has been shown to induce severe organ damage [18,19], and contact with the pesticide can induced skin irritation, nausea, diarrhea and abdominal pain [20]. In addition, unlike other xenobiotics, paraquat is not largely metabolized, and it was reported that it can reach neuronal tissue, permeating the blood–brain barrier, where its bioavailability increases with chronic exposure, leading to long-lasting effect [21,22]. When considering the nasal route, prolonged inhalation of paraquat can often induce fatal damage or long-term disorders, such as Parkinson’s disease [23]. In the European Union, paraquat use has been banned since 2007. However, due to a huge flow of import/export products from countries where paraquat is still used, its toxicity for Europeans may still arise from bioaccumulation in foodstuff [24]. Restrictions on pesticides use were hypothesized for a wide variety of chemical substances used as pesticides, as is the case of glyphosate which, in addition to being one of the most used herbicides worldwide, is also one of the most studied [25]. In this review we will discuss recent advances in the toxicity of glyphosate and its commercial formulations.

**Figure 1:**
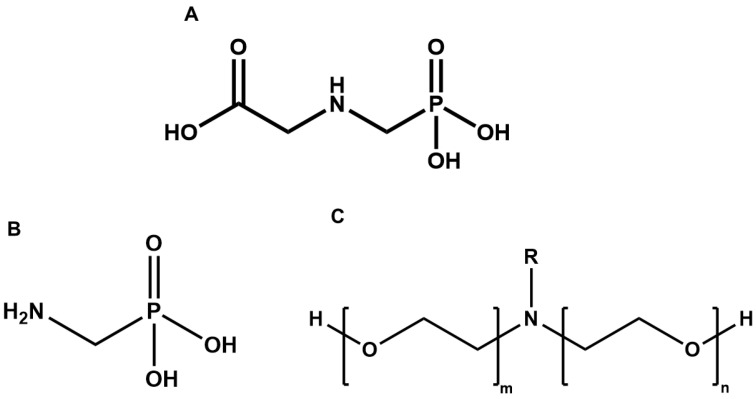
Chemical structure of (**A**) glyphosate, (**B**) the main glyphosate metabolite AMPA (aminomethylphosphonic acid) and (**C**) the common surfactant used in glyphosate-based herbicides, POEA (polyethyloxylated tallow amine).

Although it was developed in 1950 [26], glyphosate’s herbicidal activity was only described more than twenty years later, being then introduced by Monsanto in 1970. Glyphosate was sold as Roundup^®^ (Monsanto, St. Louis, MO, USA) and branded as history’s most successful herbicide, being second only to DDT (dichloro-diphenyl-trichloroethane) in overall pesticides use [27,28]. *N*-(phosphonomethyl)glycine (IUPAC nomenclature for glyphosate) is an odorless crystalline solid with a white appearance, it is derived from glycine through the addition of a phosphonomethyl group linked through the amino acid’s primary amine group, forming then a secondary amine compound (glyphosate structure is presented in Figure 1A).

Glyphosate herbicidal action is achieved through the inhibition of 5-enolypyruvylshikimate-3-phosphate synthase (EPSPS; EC 2.5.1.19), an enzyme produced and present in plants, fungi and some microorganisms, but not in animals. In those organisms, the transference of an enolpyruvyl group from phosphoenolpyruvate to shikimate-3-phosphate is catalyzed by EPSPS, in a metabolic pathway deeply involved in the biosynthesis of essential metabolites, such as amino acids (e.g., phenylalanine, tyrosine or tryptophan), and in this case impaired due to the inability to produce chorismate [29,30,31].

Glyphosate is able to form a stable complex with the enzyme, competing with phosphoenolpyruvate, and thus inhibiting EPSPS function [29,30,31]. Originally, glyphosate was widely used as a nonselective herbicide, only limited by its toxicity to the crops. This was later overcome with the introduction of glyphosate-resistant crops, namely through soybean, with a glyphosate-resistant variety introduced in 1996, whose acceptance by farmers greatly increased in the first years, leading to an even higher use of glyphosate and to the biotechnological development of new glyphosate-resistant varieties of other crops (e.g., cotton and maize) [27]. These glyphosate-resistant crops were bioengineered with the modulation of selective genes, namely *pat* and *bar* (for glufosinate), and most relevantly to glyphosate-resistance, the *cp4 epsps* gene, which encode a glyphosate-tolerant EPSPS enzyme. This resistance can be obtained by single or multiple base pair alterations in the gene, amplification/duplication of the gene, enhanced xenobiotic metabolism or others [32,33]. The primary objective is either to produce a crop in which EPSPS is present in higher concentration, or to provide increased metabolization of glyphosate into its less phytotoxic metabolites. Being the only herbicide in the market that exerted its action in EPSPS, the development of these resistant crops greatly contributed to its use [32,33]. Possible limitations to the use of glyphosate were only discussed after the first reports of its toxicity to humans, as well as its potential accumulation in soil, water or food products [34]. This toxicity was the basis for the glyphosate usage regulation imposed worldwide. 

## 2. Regulatory Measures on Glyphosate Use

The major glyphosate consumers and their respective regulatory authorities have been on an ongoing debate over glyphosate ban in recent years, where both parts present opposite classifications for the herbicide. For example, for the United States (US) and European agencies, glyphosate and its commercial formulations are classified as chemicals with low probability of being carcinogenic [34]. On the other hand, IARC’s (International Agency for Research on Cancer), has placed these substances in group 2A, as probable carcinogenic, based on their ability to induce DNA damage and oxidative stress, both hallmarks of carcinogenic compounds [34,35]. In addition, IARC’s analysis also comprises glyphosate metabolites such as aminomethylphosphonic acid (AMPA) (Figure 1B). This discussion is likely to continue, as recent reviews still point to both glyphosate and its formulations as substances of lesser preoccupation concerning genotoxicity [36]. While most regulations are defined to protect the general population concerning the concentrations of the contaminant in air, water or food, workers in charge of herbicidal application will always be exposed to a higher concentration of glyphosate, for a longer period of time and more frequently [37]. New scientific evidence of its toxicity/safety must be assessed, especially for acute and longer chronical exposures associated with worker safety.

In the European Union’s specific case, banning glyphosate has been an ongoing discussion for the past 20 years. Although already being used in a vast majority of EU countries, the herbicide was approved in 2002 and the authorization renewed in 2010, until 2015, the year in which IARC released the classification mentioned above [35]. Upon its later consideration of low probability of being carcinogenic, the European Food Safety Agency (EFSA) initiated the discussion on the use of polyethyloxylated tallow amine (POEA) (Figure 1C), a nonionic surfactant commonly used in glyphosate formulations [35]. Following glyphosate classification by EFSA, the European Chemical Agency (ECHA), stated in 2017 that glyphosate should not be classified as carcinogenic, and later that year approved for an additional five years, with the opposition of major EU members such as France and Italy [35]. Nevertheless, a proposal for glyphosate ban after 2022 was drafted, and glyphosate formulations containing POEAs were banned [35].

Other countries such as Australia, Canada or New Zealand supported the US and EU agencies classification [35,37]. The IARC decision is attributed to the fact that this agency used only peer-reviewed studies published in scientific journals, including those that examined glyphosate-based herbicides. Agencies such as EFSA, besides considering only glyphosate, as it is the active ingredient, also based their classification on manufacturers’ reports, which may not be accessible to general consulting due to confidentiality [35,37].

In this review, we discuss recent advances in glyphosate and glyphosate-based herbicides toxicity together with the role of these herbicides’ excipients in the toxicity observed and the soundness of both classifications.

## 3. Environmental Persistence of Glyphosate and AMPA

When discussing glyphosate toxicity, several factors must be taken into account. Firstly, and although many authors report glyphosate-induced toxicity, glyphosate-based herbicides are often used in those studies, whose composition usually contains only 30–50% of the herbicide, and frequently added as an isopropylamine salt, to increase the solubility, although sodium, potassium or ammonium salt derivatives may also be found [38]. In addition, as mentioned above, these formulations make use of surfactants to increase their activity, which may also contribute to the toxicity observed, and which are present in the most common formulations available, such as Roundup^®^ [39]. Thus, it is of major importance to characterize glyphosate and glyphosate-based herbicide toxicity independently. As we report below, an ever-increasing number of scientific publications are raising awareness to AMPA toxicity. AMPA is the major metabolite originating from glyphosate degradation; since its accumulation was also detected in various samples (e.g., soil, water, foodstuff, human bioaccumulation), understanding its toxicity and environmental persistence is as relevant as that of glyphosate.

Risk analysis performed by the US and European safety agencies for glyphosate were mainly regarding the general population, and thus it is relevant to analyze the residual glyphosate and AMPA concentrations to which the population is chronically exposed daily [35,37]. Published by EFSA, the conclusions on glyphosate risk assessment stated that glyphosate’s DT_50_, a measurement of a chemical compound environmental persistence as the time necessary to degrade half of its original concentration, presents high variation depending on the sample. When in soils under anaerobic conditions, glyphosate DT_50_ varies from 135 to >1000 days, thus showing high persistence, while in aerobic conditions, the laboratory studies used by EFSA presented DT_50_ ranging from 1.01 to 67.72 days, where the type of soil, pH, temperature and soil moisture were parameters also included in the analysis [40]. AMPA, on the other hand, presented higher persistence, with DT_50_ ranging from 38.98 to 300.71 days in aerobic conditions [40].

In addition to DT_50_, pesticide environmental impact is also measured as the predicted environmental concentration (PEC), namely as the PEC_accu_, which is considered a worst-case scenario relative to an application of 4.32 kg of active ingredient, by hectare, by year for ten years, in bare soil at 5 cm deep [41]. In this scenario, the PEC_accu_ obtained was 6.6 mg/kg for glyphosate, while AMPA’s PEC_accu_ was established at 6.2 mg/kg, when applying 1.527 kg of active ingredient (in the same conditions mentioned above) and considering that the maximum AMPA concentration is 53.8% of the applied dose [40].

Concerning the PEC for superficial waters (PEC_sw_) and the sediment (PEC_sed_), a single application of 4.32 kg of active ingredient by hectare was considered, resulting in a PEC_sw_ = 104.8 µg/L and PEC_sed_ = 10.3 mg/kg. Using the same application rate, AMPA’s PEC values were lower when compared with glyphosate; PEC_sw_ = 40.9 µg/L and PEC_sed_ = 3.3 mg/kg [40] were observed. EFSA’s document on glyphosate risk assessment also considers PEC_air_; however, the data analyzed gave a DT_50_ in the atmosphere of less than two days, therefore ruling out the hypothesis on long distance transport and thus considering the PEC_air_ as negligible [40].

Glyphosate presents a stable chemical structure, capable to chelate metals, and has a high persistence in soil, behaving as other common inorganic phosphates. While physical degradation is not a major intervenient, microorganisms present in both soil and water, under optimal conditions, may greatly decrease the herbicide’s half-life [42,43]. Glyphosate degradation results in either AMPA or sarcosine and glycine, depending on the degradation route taken [43]. Although glyphosate degradation is faster in water, AMPA still reveals a long half-life, ranging between 76 to 240 days, in addition to a glyphosate’s half-life that can extend up to 91 days [44]. Nevertheless, and given the array of possible soil and water conditions, other authors present different half-life times [42,43]. Others have reported a persistence in soil samples up to 180 days [38,45]. Table 1 resumes recent findings of glyphosate and AMPA accumulations in various water sources, soil, atmosphere, food products and human fluids.

Standing out as a major conclusion from Table 1, the high heterogeneity of values for both glyphosate and AMPA concentrations in the various samples can be observed. Contributing to this variety of results is the analysis methodology used, whose limits of detection, quantification and accuracy may influence the results. Currently, a wide variety of chromatographic, spectroscopic and electrochemical techniques can be applied for detecting glyphosate, reflecting an effort to provide fast and reliable quantifications of this contaminant [74]. A second factor is the geographical location of analysis as well as the sample analyzed. Although still detected in various water sources throughout Europe (ranging from less than 0.1 to 165 µg/L), these residual concentrations were significantly lower than the ones found in countries such as the US (up to 430 µg/L) [75]. This may be due to the greater use of glyphosate-resistant crops in the US, while some European countries do not allow these crops. This tendency is also observed for glyphosate presence in human urine. In the US, at least 60% of the population presented glyphosate accumulation in their urine, registering a maximum concentration of 233 µg/L of the herbicide [75], although the average value was 2 to 3 µg/L. In Europe, the measured average value was lower (<1 µg/L), as well as the maximum concentration registered (5 µg/L) [75]. Due to these different degrees of exposure between two major economic regions, their regulation concerning exposure limits is also substantially different. While the European agencies defined a daily intake of 0.5 mg/kg/day, a value based on studies using rats, which established 350 mg/kg/day as toxic based on hepatic dysfunctions, and a safe concentration of 50 mg/kg/day obtained in studies using rabbits as a model, the US agency placed their benchmark at 1.75 mg/kg/day [76]. Although the general population is generally exposed at much lower concentrations of glyphosate, some authors still characterize the admitted daily intake as too high [76,77]. In addition, once again, the effect of occupational hazard for workers involved in herbicide application is not considered. Regarding atmospheric contamination, studies for both glyphosate and AMPA persistence in air samples were also addressed. Chang et al. (2011) reported maximum glyphosate concentrations of 9.1 and 7.7 ng/m^3^ in Mississippi and Iowa, respectively [56]. Nevertheless, AMPA was detected at much lower concentrations, with average values of 0.02–0.06 ng/m^3^, and a maximum value of 0.97 ng/m^3^ [56]. As discussed above, EFSA considered PEC_air_ as negligible and a low DT_50_ [40], and thus not the main concern for general population exposure to the herbicide. However, when considering occupational hazard, glyphosate exposure through contaminated atmosphere may present higher concerns. Morshed et al. (2011) evaluated glyphosate’s concentration in the atmosphere before, during and after herbicide application [57]. While no glyphosate was detected prior to its application, in the following periods its concentration in air increased to 0.1 µg/mL (0–4 h after application) and to 0.05 µg/mL (4–8 h after application). With greater interest toward human exposure, during the application, glyphosate’s atmospheric concentration was ~43 µg/mL [57], and therefore significantly higher than the remaining values reported, which highlights the occupational hazards for workers.

As a second conclusion from analyzing Table 1, different plant products or beverages present different glyphosate content and consequently will have a different role in human diet-dependent glyphosate exposure. In particular, and due to the use of glyphosate in most crops, and the existence of glyphosate-resistant varieties, wheat, oat, corn and soybean are among the products with higher glyphosate content [58,59,60,61,62]. Naturally, this affects the glyphosate content in processed products such as bread, breakfast cereals and flours. In addition to water contamination, beverages such as beer, wine and tea also present a high content of these contaminants [61,64], most likely due to prior contamination of their raw materials. All these contribute to bioaccumulation in humans, which presuppose animals breed for human consumption can equally accumulate glyphosate and its metabolites, and therefore further increase human exposure. Additionally, with a high impact, it can be observed in Table 1 that the presence of glyphosate was reported not only in human urine, which is due to its role in xenobiotic metabolism/elimination as the primary source of analysis, but also in other human fluids such as serum [68,73]. Regarding its metabolism, glyphosate can follow mainly two routes: (i) urine elimination, as mammals are not efficient in metabolizing glyphosate, it is excreted in its original form in the urine; (ii) intestinal metabolization, as the intestinal tract microbiota may metabolize part of the glyphosate ingested into AMPA [78].

A third conclusion is that AMPA is accumulated in the same analyzed samples. Although the existing regulation is mostly directed toward glyphosate, in the case of rapid degradation, the presence of the herbicide may not be detected, and the analysis may not be taking into account the presence of AMPA. Therefore, AMPA toxicity must be thoroughly addressed.

Therefore, given the high exposure to glyphosate, its herbicidal formulations available in the market and its metabolites, a more detailed knowledge of its toxicity is mandatory. In this review we focus mainly on glyphosate, AMPA and POEA.

## 4. In Vitro and In Vivo Studies to Assess Animal Toxicity (2010–2021)

As discussed above, the decision to ban or allow glyphosate usage is shrouded in controversy. While a significant number of studies clearly validate the toxic effects of glyphosate using both in vivo and in vitro models, there are also a growing number of publications highlighting the contribution of the excipients and adjuvants present in glyphosate-based herbicides to the observed animal toxicity. Based on Table 1, it is clear that, mainly, developed countries have a generalized exposure to residual glyphosate levels (e.g., Canada [47] and Switzerland [61]). However, the actual amount of exposure and frequency is hardly trackable. Even more, as mentioned above, a glyphosate ban should also consider occupational hazard, which implies a much higher exposure when compared to the general population. Adding complexity to the subject, the published studies that address glyphosate toxicity often lack clarity in describing whether the results are relative to an analytical standard, the glyphosate content in the herbicide, or to the whole herbicide formulation. Currently, a large number of glyphosate-based herbicides can be found and used worldwide [79], with mention to more than 2000 formulations that have been identified in Europe in a single year (2012) [39]. As each formulation presents its own composition, a question is raised: To what extent can the results between formulations be compared? Table 2 presents a summary of in vivo and in vitro studies published recently that aim to assess animal toxicity induced by glyphosate, its herbicidal formulations and its metabolite AMPA.

Both glyphosate and glyphosate-based herbicide toxicity has been demonstrated over the years in various experimental models [79]. Agostini et al. (2020) published a very complete review on glyphosate-induced toxicity, using in vitro cell models. The effects observed were mostly regarding the loss of cell viability, loss of membrane integrity, genotoxicity, increased oxidative stress, lipid peroxidation and the modulation of intracellular calcium concentration ([Ca^2+^]_i_) and the cell cycle. Additionally, glyphosate can also act as an endocrine-disruptor [78].

Regarding the genotoxicity, recent findings suggest that further studies may be needed before discarding its absence. HepG2 cells (human hepatocarcinoma cell line) are frequently used as a cell model for hepatic metabolism owing to role of the liver in xenobiotic metabolism. HepG2 cells were exposed to glyphosate at concentrations between 0.5 and 3.5 µg/mL, aiming to compare concentration within the range of admitted daily intake, residential exposure and occupational exposure [80]. No negative impact was observed in cell viability [80]. In fact, a slightly higher cell viability was observed in cells exposed to the herbicide, supported by control-like oxidative stress markers (reactive oxygen species level and glutathione content), and no DNA damage was observed, as assessed by comet assay [80]. However, the authors observed an increased formation of micronuclei, and an asynchrony of the cell cycle phase compared to the control cells [80]. Other authors observed genotoxicity to glyphosate in different models, but only at higher concentrations [80,81]. However, even at low concentration there is the possibility of covalent adducts formation. The formation of these adducts by some compounds lead to DNA’s interstrand cross-links, which can cause bending and unwinding. As the cell cycle progresses, these modifications may impair DNA replication, and further cause cell cycle arrest and/or apoptosis, while not manifesting major signs of genotoxicity [80].

In addition to studies on human cell lines, the effect of glyphosate-based herbicides, their active ingredient and excipients has been fairly well described in a wide variety of animal models, as seen in Table 2. From common models used in animal experimentation, such as the Wistar rat, fruit fly (*Drosophila melanogaster*), zebrafish (*Danio rerio*), water flea (*Daphnia magna*) and the roundworm (*Caenorhabditis elegans*), which are frequently used for later extrapolation to humans in studies of genotoxicity, neurotoxicity and tumor studies, to lesser-known species such as the Pacific oyster (*Crassostrea gigas*) or the common toad (*Bufo spinosus*), a wide variety of effects have been observed.

Corroborating the data reviewed by Agostini et al. (2020) in human cell lines, animal models subjected only to glyphosate also exhibited toxic features, mostly measured as morphological or general well-being parameters such as weight, mobility, reproduction, or feed intake [78]. Only a few studies consider metabolic biomarkers such as oxidative stress or hormonal modulation. The lack of evidence regarding the metabolic pathways involved in glyphosate toxicity still requires extensive study in in vivo models. In weaned pigs, a recent study reported glyphosate’s toxic effect on intestinal epithelium morphology and barrier function [82]. However, closer analysis of the published study once again revealed that the authors used Roundup^®^, with only 30% of its content being glyphosate, and thus, although expressing the results as mg of glyphosate/kg of diet, other excipients were present in the mixture feed to the animal model. Nevertheless, the authors verified that the herbicide modulated the expression of tight-junction proteins at intestinal level, verified as reduced mRNA expression of both tight junction protein-1 (ZO-1) and claudin-1 [82]. Additionally, oxidative stress markers were altered, with an increase in nuclear factor erythroid 2-related factor 2 (Nrf2) expression, a protein involved in oxidative stress response. In addition, the authors reported an increased expression of inflammation markers, namely interleukin-6 (IL-6) and nuclear factor kappa-light-chain-enhancer of activated B cells (NF-kB), but had no effect on IL-1β and IL-8 mRNA expression [82].

**Table 2:** Recent scientific publication on glyphosate, glyphosate-based herbicides and AMPA toxicity in animal cell cultures and animal experimental models.

	Model	Exposure Time	Tested Concentrations	Effects	Ref.
Glyphosate (99.8%)	*Cherax quadricarinatus*	60 days	10 and 40 mg/L	Decrease in lipid levels in muscle, as well as protein level in hepatopancreas and muscle	[83]
Glyphosate (99%)	*Danio rerio*	96 h	1.7–100 mg/L	Genotoxicity, morphological abnormalities	[84]
Glyphosate (˃98%)	Hormone-dependent breast cancer (T47D cell line)	24 h	10^−9^–10^−3^ mM	Increase in cell proliferation	[85]
Glyphosate (95%)	Human keratinocytes (HaCaT cell line)	24 h	10–70 mM	Loss of cell integrity, overproduction of H_2_O_2_, membrane damage, apoptosis induction, genotoxicity	[86]
Glyphosate (95%)	Buccal epithelial cells (TR146 cell line)	20 min	>10 mg/L	Increased lactate dehydrogenase release, DNA damage	[87]
Glyphosate (99%)	Human hepatocarcinoma (HepG2 cell line)	4 and 24 h	0.5–3.5 µg/mL	Micronuclei formation, lower antioxidant capacity	[80]
Glyphosate (90%)	Sprague Dawley rat	5 weeks	5–500 mg/kg	Decreased average daily feed intake and decreased total sperm count	[88]
Glyphosate (not specified)	Induced pluripotent stem cells (iPSCs)	24 h	1–1000 µM	Increase in blood–brain barrier permeability to fluorescein, changes in neuronal cells metabolic activity and increase of glucose uptake in brain’s microvascular endothelial cells	[89]
Glyphosate (40%)	*Daphnia magna*	60 days	0.5–4.05 mg/L of glyphosate	Reduction of juvenile’s size, decreased fecundity and longevity	[90]
Glyphosate (not specified)	*Danio rerio*	21 days	10–100 mg/L of glyphosate	Reduced egg production, increase in early-stage embryo mortalities and premature hatching, disruption of the steroidogenic biosynthesis pathway, oxidative stress	[91]
Glyphosate (not specified)	*Danio rerio*	48 h	50 µg/mL of glyphosate	Structural abnormalities in the atrium and ventricle, irregular heart looping, situs inversus and decreased heartbeats	[92]
Glyphosate (not specified)	*Danio rerio*	15 days	65 µg/mL of glyphosate	Increase in oocytes’ diameter, presence of concentric membranes appearing as myelin-like structures, increase in expression of SF-1 in oocytes	[93]
Glyphosate (not specified)	*Danio rerio*	96 h	0.01–0.5 mg/L of glyphosate	Decrease locomotion in adult zebrafish, decreased ocular distance in zebrafish larvae	[94]
Glyphosate (not specified)	Sprague Dawley rat	2 weeks	50–150 mg/kg of glyphosate	Hypoactivity, decrease in specific binding to D1 dopamine receptors in the nucleus accumbens, decrease in basal extracellular dopamine levels and high-potassium-induced dopamine release in striatum	[95]
Roundup (480 g/L)	*Piaractus mesopotamicus*	48 h	3.0–4.5 mg /L of glyphosate	Cytoplasmic vacuolization, lipid accumulation, nuclear and cellular membrane alterations and glycogen depletion in the liver	[96]
Touchdown^®^ (523 g/L)	*Caenorhabditis elegans*	30 min	3–10% of glyphosate	Inhibition of mitochondria’s complex II, decrease in ATP levels, increase in H_2_O_2_ levels	[97]
Roundup (410 g/L)	Human alveolar carcinoma (A549 cell line)	2 h	100 µg/L	Inhibition of cell proliferation, collapse of mitochondrial membrane, oxidative DNA damage, DNA single-strand breaks and double-strand breaks	[98]
Roundup (180 g/L)	*Daphnia magna*	60 days	0.5–4.05 mg/L of glyphosate	Reduction of juvenile size, growth, fecundity and increased abortion	[90]
Roundup (180 g/L)	*Drosophila melanogaster*	24 h	15 µg/mL	Decreased lifespan, fecundity, cell viability of ovarian sheath cells, negative geotaxis response, increase in protein carboxyl levels and enhanced caspase activity indicative of pro-apoptotic process	[99]
Herbolex (486 g/L)	*Daphnia magna*	48 h	20–137 µg/L	Increased lipid peroxidation, feed inhibition, increase in antioxidant enzyme activity	[100]
Roundup (480 mg/L)	*Poecilia reticulata*	96 h	0.34–5.2 mg/L of glyphosate	Modulation of energy and nucleic acids metabolism, cytoskeleton and proteins; progressive histopathological damage in the gills	[101]
Roundup (450 g/L)	Buccal epithelial cells (TR146 cell line)	20 min	>10 mg/L of glyphosate	Increase in nucleoplasmatic bridges, nuclear aberrations and micronuclei	[87]
Roundup (360 g/L)	Albino rats	12 weeks	3.6–248.4 mg/kg of glyphosate	Accumulation of glyphosate residue in kidney tissue, histopathological lesions in kidneys, distorted renal cortical histoarchitecture, expanded urinary space due to glomerulosclerosis, and tubular necrosis	[102]
Roundup (360 g/L)	Mice	6 and 12 weeks	250 or 500 mg/kg/day	Decrease in body weight gain and locomotor activity, increase of anxiety and depression-like behavior levels	[103]
Roundup (360 g/L)	Albino rats	12 weeks	3.6–248.4 mg/kg/day of glyphosate	Decrease in the mean level of testosterone, FHS and LH in the blood, and increase of prolactin, excessive production of ROS, reduction in sperm count, percentage mobility and increase in abnormal sperm cells, degenerative testicular lesions	[104]
Roundup (360 g/L)	*Anguilla anguilla*	1 and 3 days	18 and 36 µg/L	Increment of catalase activity in gills, decrease of superoxide dismutase activity in liver, increase in DNA damage	[105]
Roundup (360 g/L)	Murine Sertoli cells (TM4 cell line)	24 h	10–10,000 mg/L	Decrease of succinate dehydrogenase activity, inhibition of glutathione-*S*-transferase, disruption of cell detoxification systems, increase of cytoplasmatic lipid droplets	[106]
Roundup (360 g/L)	*Colossoma macropomum*	96 h	10 and 15 mg/L of glyphosate	Alterations in respiratory epithelium structure, changes in hematological parameters, increase ROS production, increase in DNA damage in red blood cells and inhibition of cholinesterase activity in fish brain	[107]
Roundup (120 g/L)	*Danio rerio*	21 days	0.01–10 mg/L of glyphosate	Increase in early-stage embryo mortalities and premature hatching, disruption of the steroidogenic biosynthesis pathway, oxidative stress	[91]
Roundup (120 g/L)	*Daphnia magna*	48 h	100–300 mg/L	Loss of whole body enzyme activity and loss of cells integrity	[108]
Roundup	*Danio rerio*	96 h	0.01–0.5 mg/L	Decrease in locomotion in adult zebrafish, ocular distance in zebrafish larvae and decrease in aggressive behavior in adult zebrafish, impairment in memory in adult zebrafish	[94]
AMPA	*Daphnia magna*	21 days	7.4–120 mg/L	Decreased neonate production	[109]
AMPA	*Bufo spinosus*	16 days	0.07–3.6 µg/L	Decrease in embryonic survival, development delay, modification of body morphology	[110]
AMPA	*Danio rerio*	24–96 h	1.7–100 mg/L	Genotoxicity, morphological abnormalities	[84]
AMPA	Induced pluripotent stem cells (iPSCs)	24 h	0.1–1000 µM	Increase in blood–brain barrier permeability to fluorescein, changes in neuronal cells metabolic activity and glucose uptake in brain microvascular endothelial cells	[89]
AMPA	Human erythrocytes	4 and 24 h	0.01–5 mM	Increased ROS production, hemolysis and hemoglobin oxidation	[111]
AMPA	*Paracentrotus lividus*	24 and 48 h	1–100 µg/L	Development delay, increase of respiration rate, reduction in larvae size	[112]

Notes: Glyphosate content in glyphosate-based herbicides is denoted as percentage under the formulation name. For studies regarding glyphosate-based herbicides where the authors calculated and expressed their results as the glyphosate concentration used, are listed as, for example, “mg/L of glyphosate”, while studies mentioning, for example, “mg/L” are relative to the concentration of the whole glyphosate-based herbicide.

Additionally worthy of discussion, as seen in Table 2, AMPA and glyphosate share a large number of effects in the animal models tested, from the more common effects associated with genotoxicity [84] and oxidative stress [111], to morphology and fecundity issues [110]. AMPA toxicity to humans is still largely understudied. Recent studies still do not present sufficient scientific evidence to justify a major discussion of AMPA exposure, bioaccumulation and toxicity that could lead to further legislation on its detection and contribute to a glyphosate ban.

## 5. Toxic Effect of Surfactants Used in Glyphosate-Based Herbicides

The toxicity of the excipients and adjuvants that compose glyphosate-based herbicides has also been addressed, as these components often present higher toxicity to the nontarget species than the active compound. Concerning POEAs (polyethoxylated tallow amine), present, for example, in the main glyphosate-based herbicide Roundup^®^, the name represents a class of nonionic surfactants where the amine moiety (Figure 1C) is lipid-based, namely from animal fat, and thus referred as tallow (a mixture of amines derived from palmitic acid (C16, saturated), oleic acid (C18, mono-unsaturated), stearic acid (C18, saturated) and others) [113]. Apart from this, the molecules also contain two chains of ethylene oxides, being therefore referred as polyethyloxylated tallow amines. New generations of herbicide formulations with non-POEA surfactants have been proposed as an alternative, following the EU ban, a regulation that was not adopted by the US, and thus keeping the discussion of POEAs toxicity a matter of general interest [39]. Just as described for glyphosate and glyphosate-based formulations, effects on the experimental models’ fecundity, genotoxicity and overall toxicity have been described for POEAs [84,99,106,114,115], although less extensively. This deserves particular attention, since, as seen in Table 2, some authors report their results based either on the concentration of glyphosate in the herbicide formulation, or the whole herbicide formulation concentration, there are even studies that do not specify what the indicated concentration refers to (if the active compound or the formulation), which complicates the comparison between results [39]. Table 3 presents recent studies regarding POEAs toxicity observed in in vivo and in vitro experimental methods.

Using *Drosophila melanogaster* as an in vivo model, Bednářová et al. (2020) have contributed to a new insight in glyphosate vs. glyphosate-based herbicide toxicity [99]. Briefly, the authors analyzed Roundup^®^ Concentrate Plus (143 g/L of glyphosate isopropylamine salt) toxicity in this model, verifying that at 15 µg/mL, the flies’ lifespan and fecundity were reduced, when the same was not observed for glyphosate at 100 µg/mL. While glyphosate presented a LC_50_ of 5146 μg/mL, Roundup^®^ presented a LC_50_ value of 774.4 μg/mL [99]. To acquire information about the toxicity of the excipients, the authors also tested a POEA, whose LC_50_ was 1322.6 μg/mL, and thus much lower than that of glyphosate. In addition, both Roundup^®^ and POEA increased protein carbonyl concentration and decreased carbonyl reductase activity, both biomarkers of oxidative damage in proteins, contrarily to glyphosate, which did not alter protein carbonyls levels [99].

Similar findings were observed using an in vitro cell model of murine Sertoli cells (TM4), using glyphosate and two glyphosate-based herbicides [106]. While glyphosate had no effect on cell viability after 24 h exposure, for the same concentration both formulations induced dose-dependent cytotoxicity, with reduced glutathione-*S*-transferase activity. The authors also observed lipid droplet accumulation only with formulated glyphosate. To ascertain the potential effect of POEAs in this toxicity, the authors evaluated the effect of POE-15, confirming its role in lipid droplet accumulation and suggesting the bioaccumulation of the surfactant in the cells. Even after a small incubation period (2 min), to 0.5% of POE-15, a great reduction in cell viability was observed. Exposure of TM4 cells, for 24 h, to POE-15 at a concentration of 0.01%, revealed to be sufficient to induce 100% cell death [106].

**Table 3:** Evaluation of POEA toxicity using in vivo and in vitro experimental models.

Model	Exposure Time	Tested Concentrations	Effects	Ref.
Wistar rat	15 min	1.28–800 mg/L	Disturbances of the spontaneous motoric activity of isolated jejunum segments	[114]
*Crassostrea gigas*	35 days	0.1–100 µg/L	Delay in gametogenesis, connective tissue destructuration, atrophies of the wall of digestive tubules	[115]
*Danio rerio*	24–96 h	0.4–16 mg/L	Genotoxicity, morphological abnormalities	[84]
Murine Sertoli cells (TM4 cell line)	24 h	>0.01%	High cytotoxicity (0% cell viability)	[106]
*Drosophila melanogaster*	24 h	45 µg/mL	Decrease in lifespan, negative geotaxis response, increase in protein carboxyl levels, decrease in fecundity, decrease of ovarian sheath cells viability, enhanced caspase activity indicative of pro-apoptotic process	[99]

In humans diagnosed with severe systemic toxicity due to glyphosate ingestion, the analysis of the various cases in Korean hospital records (*n* = 107) resulted in a major conclusion, that the amount of glyphosate-based herbicide ingested was the determi-nant for observed toxicity and correlated with the volume of ingested surfactant, inde-pendent of the surfactant. The patients exhibited symptoms ranging from hyperten-sion, arrhythmia, respiratory failure and renal trauma. In two of the patients, metabolic acidosis, respiratory failure and refractory shock culminated in death [116].

Nevertheless, given the volume of excipients in herbicide formulations, not only glyphosate or POEAs may be considered either safe or the only toxic component. Ad-ditionally, the excipients should be inert; however, this is not the case, since petrole-um-based compounds, arsenic, lead, cobalt or polycyclic aromatic hydrocarbons are of-ten present in pesticide formulations [117,118]. In fact, some of these compounds pre-sent higher toxicity than glyphosate, and the possible synergistic interactions between compounds may contribute to an increase in the toxicity of glyphosate formulations, which is not so severe when glyphosate is applied as a standard molecule rather than a formulation alone. In addition, some of these components may not be declared in the composition list, thus raising questions regarding the source of toxicity [117,118].

## 6. Conclusions 

Glyphosate toxicity has been the target of an ongoing debate, in part powered by a lack of clarity in the data published; some studies use only glyphosate, others use formulations containing glyphosate and a series of non-discriminated compounds. Glyphosate is regarded as the most successful herbicide in history; however, assurance of its safety at the current exposure concentrations for the general population still requires a larger number of scientific publications that support a decision. First, the actual exposure should be accurately defined and take in account soil, water, air and food contamination. Second, glyphosate-based herbicide compositions should be accurately described for each formulation, so their toxicity can be correctly analyzed based on both glyphosate content and the excipients present. Nevertheless, the toxicity of such formulations is currently well proved, and requires a revision of the regulation. Efforts should be made to better regulate the production of these formulations, their use and, therefore, the environmental contamination. In addition, new strategies to minimize the environmental impact and toxicity to animals by these pesticides are under investigation, which should be seen as a promising strategy to insure the safe use of these products.

## Figures and Tables

**Table 1:** Concentration of glyphosate and of its metabolite, AMPA, in various sources with relevance for animal toxicity.

Compound	Sample	Concentration	Detection Method	Ref.
Glyphosate	Rainwater	6.1 µg/L	LC–MS^n^	[46]
	Stream water	41 ng/L	IC/MS^n^	[47]
	Groundwater	4 µg/L	LC–MS	[48]
	Groundwater	21.2 µg/L	UHPLC–MS^n^	[49]
	Groundwater	0.025 µg/L	LC–MS^n^	[50]
	Lake water	4.52 µg/L	HPLC–MS^n^	[51]
	Lake water	45 µg/L	LC–MS	[48]
	Marine water	1.7 µg/L	LC–MS^n^	[52]
	Suspended particulate matter	584 µg/kg	UHPLC–MS^n^	[53]
	Water	17 µg/L	UHPLC–MS^n^	[54]
	Suspended particulate matter	0.13 µg/L	HPLC–MS^n^	[51]
	Sediment	20.34 µg/kg	HPLC–MS^n^	[51]
	Sediment	3294 µg/kg	UHPLC–MS^n^	[53]
	Sediment	1000 µg/kg	LC–MS	[48]
	Soil	8105 µg/kg	UHPLC–MS^n^	[53]
	Soil	1502 µg/kg	UHPLC–MS^n^	[55]
	Soil	690 µg/kg	LC–MS	[48]
	Air	0.48 ng/m^3^	HPLC-MS	[56]
	Air	0.24 ng/m^3^	HPLC-MS	[56]
	Air (application)	42.96 µg/m^3^	HPLC-FD	[57]
	Air (0–4 h after application)	0.1 µg/m^3^	HPLC-FD	[57]
	Air (4–8 h after application)	0.05 µg/m^3^	HPLC-FD	[57]
	Organic oat flour	11 µg/kg	LC–MS^n^	[58]
	Oatmeal	1100 µg/kg	LC–MS^n^	[58]
	Oat-based cereals	901 µg/kg	LC–MS^n^	[58]
	Oat flour	554 µg/kg	LC–MS^n^	[58]
	Wheat	670 µg/kg	LC–MS^n^	[59]
	Durum wheat	421 µg/kg	LC–MS^n^	[60]
	Breakfast cereal	291 µg/kg	LC–MS^n^	[61]
	Soy protein isolate	105 µg/kg	UHPLC–MS^n^	[62]
	Soy protein concentrate	850 µg/kg	UHPLC–MS^n^	[62]
	Soybean	8800 µg/kg	HPLC-FD	[63]
	Corn	1.6 µg/kg	ELISA kit	[64]
	Coffee	26.32 µg/kg	ELISA kit	[64]
	Pea	60 µg/kg	LC–MS^n^	[59]
	Wine	18.9 µg/kg	LC–MS^n^	[61]
	Beer	2.8 µg/kg	ELISA kit	[64]
	Tea leaves	40.43 µg/kg	ELISA kit	[64]
	Tea bag	728.2 µg/kg	ELISA kit	[64]
	Bread	45.8 µg/kg	LC–MS^n^	[61]
	Honey	220 µg/kg	HPLC-FD	[65]
	Honey	49.8 µg/kg	LC–MS^n^	[66]
	Cat and dog food	0.03 µg/kg	ELISA kit	[67]
	Human urine	7.4 µg/L	LC–MS^n^	[68]
	Human urine	1.36 µg/L	LC–MS^n^	[69]
	Human urine	7.2 µg/L	LC–MS^n^	[70]
	Human urine	5.6 µg/L	LC–MS^n^	[71]
	Human urine	3.3 ng/L	ELISA kit	[72]
	Human serum	1477 µg/mL	LC–MS^n^	[73]
	Human serum	89 µg/mL	LC–MS^n^	[73]
AMPA	Rainwater	5.8 µg/L	LC–MS	[46]
	Groundwater	6.5 µg/L	UHPLC–MS^n^	[49]
	Groundwater	0.65 µg/L	LC–MS^n^	[50]
	Groundwater	11 µg/L	LC–MS	[48]
	Lake water	0.90 µg/L	HPLC–MS^n^	[51]
	Marine water	4.2 µg/L	LC–MS^n^	[52]
	Water	4.5 µg/L	UHPLC–MS^n^	[54]
	Suspended particulate matter	475 µg/kg	UHPLC–MS^n^	[53]
	Suspended particulate matter	0.07 µg/L	HPLC–MS^n^	[51]
	Sediment	7219 µg/kg	UHPLC–MS^n^	[53]
	Sediment	15 µg/kg	LC–MS	[48]
	Sediment	32.89 µg/kg	HPLC–MS^n^	[51]
	Soil	38,939 µg/kg	UHPLC–MS^n^	[53]
	Soil	2256 µg/kg	UHPLC–MS^n^	[55]
	Soil	8 µg/kg	LC–MS	[48]
	Air	0.06 ng/m^3^	HPLC-MS	[56]
	Air	0.02 ng/m^3^	HPLC-MS	[56]
	Oatmeal	40 µg/kg	LC–MS^n^	[58]
	Oat-based cereals	25 µg/kg	LC–MS^n^	[58]
	Oat flour	25 µg/kg	LC–MS^n^	[58]
	Breakfast cereal	10 µg/kg	LC–MS^n^	[61]
	Durum wheat	247 µg/kg	LC–MS^n^	[60]
	Soy protein isolate	210 µg/kg	UHPLC–MS^n^	[62]
	Soy protein concentrate	2710 µg/kg	UHPLC–MS^n^	[62]
	Soybean	10,000 µg/kg	HPLC-FD	[63]
	Wine	3.4 µg/kg	LC–MS^n^	[61]
	Honey	100 µg/kg	HPLC-FD	[65]
	Honey	50.1 µg/kg	LC–MS^n^	[66]
	Human urine	1.53 µg/L	LC–MS^n^	[69]
	Human serum	1.5 µg/mL	LC–MS^n^	[73]
	Human serum	0.07 µg/mL	LC–MS^n^	[73]

Abbreviations: HPLC, high-performance liquid chromatography; UHPLC, ultra high-performance liquid chromatography; LC, liquid chromatography; IC, ion chromatography; MS, mass spectrometry; FD, fluorescence detector.
